# Silicon Effects on Biomass Carbon and Phytolith-Occluded Carbon in Grasslands Under High-Salinity Conditions

**DOI:** 10.3389/fpls.2020.00657

**Published:** 2020-05-26

**Authors:** Linan Liu, Zhaoliang Song, Changxun Yu, Guanghui Yu, Rob M. Ellam, Hongyan Liu, Bhupinder Pal Singh, Hailong Wang

**Affiliations:** ^1^School of Environmental Science and Engineering, Tianjin University, Tianjin, China; ^2^Institute of Surface-Earth System Science, Tianjin University, Tianjin, China; ^3^Department of Biology and Environmental Science, Linnaeus University, Kalmar, Sweden; ^4^Scottish Universities Environmental Research Centre, East Kilbride, United Kingdom; ^5^College of Urban and Environmental Sciences, Peking University, Beijing, China; ^6^School of Environmental and Rural Science, University of New England, Armidale, NSW, Australia; ^7^NSW Department of Primary Industries, Elizabeth Macarthur Agricultural Institute, Menangle, NSW, Australia; ^8^School of Environmental and Chemical Engineering, Foshan University, Foshan, China; ^9^Key Laboratory of Soil Contamination Bioremediation of Zhejiang Province, Zhejiang A & F University, Hangzhou, China

**Keywords:** carbon cycle, grassland species, phytolith-occluded carbon, salinity stress, silicon cycle

## Abstract

Changes in climate and land use are causing grasslands to suffer increasingly from abiotic stresses, including soil salinization. Silicon (Si) amendment has been frequently proposed to improve plant resistance to multiple biotic and abiotic stresses and increase ecosystem productivity while controlling the biogeochemical carbon (C) cycle. However, the effects of Si on plant C distribution and accumulation in salt-suffering grasslands are still unclear. In this study, we investigated how salt ions affected major elemental composition in plants and whether Si enhanced biomass C accumulation in grassland species *in situ*. In samples from the margins of salt lakes, our results showed that the differing distance away from the shore resulted in distinctive phytocoenosis, including halophytes and moderately salt-tolerant grasses, which are closely related to changing soil properties. Different salinity (Na^+^/K^+^, ranging from 0.02 to 11.8) in plants caused negative effects on plant C content that decreased from 53.9 to 29.2% with the increase in salinity. Plant Si storage [0.02–2.29 g Si m^–2^ dry weight (dw)] and plant Si content (0.53 to 2.58%) were positively correlated with bioavailable Si in soils (ranging from 94.4 to 192 mg kg^–1^). Although C contents in plants and phytoliths were negatively correlated with plant Si content, biomass C accumulation (1.90–83.5 g C m^–2^ dw) increased due to the increase of Si storage in plants. Plant phytolith-occluded carbon (PhytOC) increased from 0.07 to 0.28‰ of dry mass with the increase of Si content in moderately salt-tolerant grasses. This study demonstrates the potential of Si in mediating plant salinity and C assimilation, providing a reference for potential manipulation of long-term C sequestration via PhytOC production and biomass C accumulation in Si-accumulator dominated grasslands.

## Introduction

Grasslands, which cover almost 40% of Earth’s land surface, play a significant role in the biogeochemical cycles of many elements, including carbon (C) and silicon (Si) (Rumpel et al., 2015; Klotzbücher et al., 2018b). Most grasses, especially the Poaceae, are hyper-Si-accumulators with the silica content ranging from less than 1% to more than 10% of dry weight (dw) (Song et al., 2012a). Grassland productivity controls not only plant biomass C accumulation and soil C sequestration (Chou et al., 2008; Xi et al., 2016), but also the coupled terrestrial Si and C cycles. However, climate change and environmental stresses can alter vegetation types and species abundance, thereby restricting C fixation and influencing Si distribution in plants by affecting the primary (i.e., carbohydrate, nitrogen, and polyol) and secondary (i.e., phenolic, flavonol glycosides, and phytohormone) metabolisms (Kuokkanen et al., 2001; Garamvölgyi and Hufnagel, 2013; Ode et al., 2014; Johnson and Hartley, 2018). After drought, soil salinization is the second limiting factor for plant growth and biomass productivity in arid and semi-arid grasslands (Conde et al., 2011; Bui, 2013). Sodium (Na) accumulation in plant shoots and leaves results in reduction of photosynthetic C assimilation and plant productivity due to oxidative stress to the photochemical apparatus (e.g., chloroplast, photosynthetic pigments, or light electron transport) (López-Berenguer et al., 2008; Rios et al., 2017).

Silicon is primarily taken up and transported in plants in the form of monomeric silicic acid (H_4_SiO_4_), subsequently polymerized to silica gel nanoparticles and deposited in plant tissues in the form of phytoliths, serving as a mechanical barrier against environmental stresses (Ma et al., 2007; Klančnik et al., 2014; Amin et al., 2016). Silica deposition in plant leaves contributes to improvement of light capture via strengthening cell membrane integrity and leaf blades, and thus enhances photosynthetic assimilation of carbon dioxide (CO_2_) and accumulation of biomass C under various biotic and abiotic stress conditions (Li et al., 2018; Wang Y. W. et al., 2019). Recently, some researchers have reported a trade-off strategy between Si and C components in Si-accumulating species under sub-optimal conditions. Silica in plant tissues plays an important role in strengthening plant erectness, especially for the wetland species, with a functional effect that is similar to C-based structural components such as lignin and phenolics (Frew et al., 2016; Johnson and Hartley, 2018). Therefore, Si components in plant tissues tend to substitute partly for C-based components to provide mechanical structural support. This trade-off phenomenon provides innovative perspectives on plant acclimation and is evidence for coupled Si-C cycling. Despite the fact that Si has been reported to enhance plant biomass via improving physical and biochemical processes (i.e., osmotic regulation, nutrient uptake, and photosynthetic strengthening) (Alzahrani et al., 2018; Javaid et al., 2019; Johnson et al., 2019; Zargar et al., 2019), the effects of Si on plant C accumulation in salinized grasslands have not been evaluated *in situ*.

Phytoliths can occlude organic carbon (OC) compounds during their formation process that amorphous silica deposits into the cell walls or fills in the cell lumen, and environmental conditions can affect the chemistry and isotopic composition of phytoliths (Hodson et al., 2008; Hodson, 2016; Yang et al., 2018). Based on the stable C isotope and radiocarbon composition of plant phytoliths, the major source of phytolith-occluded C (PhytOC) is plant-accumulated C via photosynthetic assimilation from atmospheric CO_2_ (Zuo et al., 2017). Although PhytOC accounts for a small portion (∼1.5% for Poaceae) of biomass C in plants (Song et al., 2016), it can be preserved in soils or sediments over thousands of years (Anala and Nambisan, 2015; Hodson, 2016). The high content of Si and high above-ground net primary productivity (ANPP) in grasslands would play important roles in sequestration of atmospheric CO_2_ via PhytOC production, especially for the Si-accumulating Poaceae and Cyperaceae (Song et al., 2012a; Ru et al., 2018). PhytOC concentration in plants correlates highly with Si accumulation and phytolith development, which are influenced by growth conditions, such as moisture and temperature that are closely related to plant transpiration (Buján, 2013; Meunier et al., 2017). The micro-structures of phytoliths including morphological sizes or specific surface area, which might play an important role in OC occlusion in phytoliths (Li et al., 2020), vary with species and/or plant tissues. Wang C. et al. (2019) found that the morphological sizes of bulliform phytoliths in rice were likely related to environmental factors including temperature, moisture and precipitation. In addition, soil pH in different habitats caused alteration of phytolith sizes and contents in *Leymus Chinensis*, which might result from the change of cell space in plant leaves induced by stimulating photosynthesis along with an increase in soil pH (Jie et al., 2010). However, whether plant salinity level (Na^+^/K^+^ ratio) can affect PhytOC production and whether this might be controlled by bioavailable Si in grasslands are still unclear (Deléglise et al., 2015; Harpole et al., 2016).

Most previous studies are concerned with whether Si supplementation increases plant growth and biomass productivity under stressed conditions (i.e., drought, salt, and heavy metal) (Detmann et al., 2012; Daoud et al., 2018; Li et al., 2018; Ahmad et al., 2019; Khan et al., 2020). However, less is known about the relationships between Si and C contents, Si accumulation and biomass C accumulation in plants, especially for Si-dominated species in natural fields. It is hypothesized that: (i) due to the trade-off strategy between Si- and C-based defense in Si-accumulating grasses under adverse conditions, there would usually be a negative correlation between Si and C contents in plants, which is vital for understanding the coupled Si-C terrestrial cycling associated with environmental change in soil-plant systems; and (ii) soil bioavailable Si is one of the factors associated positively with Si storage and C assimilation in plants, but negatively with plant salt ion accumulation. These hypotheses provide a reliable basis for investigating the role of Si in regulating C fixation and biomass C accumulation due to alleviation of plant salt stress. The objectives of this study were (i) to better understand how Si affects the C cycle of grasslands *in situ* under high but variable salinity conditions; and (ii) to provide implications for grassland management based on the accumulation of biomass C in plants via regulating soil bioavailable Si and even the sequestration of long-term C by increasing of PhytOC production.

## Materials and Methods

### Study Sites and Sample Collection

**FIGURE 1:**
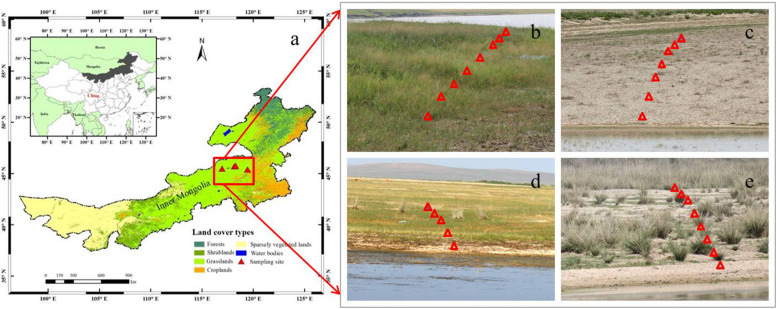
Locations of the study region in Inner Mongolia in northern China **(a)**, and the sampled scenes that are showing plant communities around the individual lakes **(b–e)**. The solid red triangles represent the selected sampling grasslands around four salt lakes **(a)**, and the hollow red triangles represent the eight quadrats (1 m × 1 m) in one of three transects in the individual sampling grasslands **(b–e)**.

The steppe district of Inner Mongolia is one of the most important native rangelands of China, covered with large areas of grasslands mainly including meadow steppe, typical steppe and desert steppe, which account for almost 80% of the entire state-owned land area (Han et al., 2008). The transects of this study mainly lie at Xilin Gol League, northeastern Inner Mongolia, China (43–45°N, 113–119°E) (Figure 1). This area has a continental semi-arid climate. The mean annual temperature is 1.5°C but with a high average annual temperature variation of more than 35°C and the maximum temperature above 40°C in summer. There is also a high diurnal temperature variation (≈15°C) and long hours of sunshine (up to 9 h) in summer, which leads to a high ANPP in grasslands due to the high photosynthetic rate (Mao et al., 2014). However, the low precipitation (≈330 mm) and relatively high evaporation (≈2,000 mm) induce differing degrees of water deficit and soil salinity to grasses.

In order to investigate the relationships between Si and C accumulation within plants, as well as soil bioavailable Si and plant biomass C in grasslands with salinity conditions *in situ*, four grasslands around salt-lakes were selected and investigated in July, 2016. Based on the field investigation, these grasslands are considered to be suffering from different levels of salinity stress, resulting in variable vegetation growth and distribution. Due to high salinity in the lake, few species except for halophytes can tolerate the habitats closest lakeshores (about 0–100 m away from the lakeshore). At the outer sites (about 100–500 m away from the lakeshore), some species that are tolerant to salinity stress can survive with different biomass productivity. These regions (within 500 m from the lakeshore) are rarely impacted by human and animal activities at least in recent years because of the poorer pasture growth than the other regions (>500 m away from salt-lake shores), which are less affected by salinity stress.

At each grassland surrounding the salt-lakes, three transects in different directions were selected based on plant growth conditions and the topographical availability. At each transect, eight quadrats (1 m × 1 m) were arranged roughly perpendicular to the lakeshore, i.e., the first (most proximal) site was closest to the water’s edge (about 0–100 m). In contrast, the outermost (distal) sites were located at sufficient distances (about 100–500 m) from the edge to have enough plant samples including halophytes and moderately salt-tolerant species growing under decreasing degrees of salinity conditions (Zhao et al., 2011; Table 1). We collected the dominant plant species in each quadrat during the maturation stage before harvesting at the end of July, during which the grasses usually accumulate the largest amount of Si and biomass C on a yearly basis (Klančnik et al., 2014). We packed each of the plant species separately in paper bags and transported them back into the lab for chemical analysis and phytolith extraction. Meanwhile, three topsoil (0–20 cm) samples from each quadrat were collected using a cutting ring method and mixed as one composite sample, which was then packed in a ziplock bag and taken to the lab for physicochemical analysis.

**TABLE 1:** Primary properties of soil and plant types in the corresponding sampling regions.

	Soils					
Distance^1^ (m)	Bulk density (g cm^–3^)	pH	EC^2^ (mS cm^–1^)	Moisture (%)	Si_*CaCl*2_ (mg kg^–1^)	Dominant species
0–100	1.46 ± 0.19a	10.1 ± 0.28a	2.66 ± 1.65a	12.8 ± 4.77a	128 ± 19.3b	*Kalidium foliatum*
(*n* = 15)						*Achnatherum splendens*
100–500	1.37 ± 0.19a	9.52 ± 0.40b	0.90 ± 0.56b	7.41 ± 3.44b	147 ± 17.8a	*Leymus chinensis*
(*n* = 36)						*Arenariae radix*
						*Achnatherum splendens*
						*Agropyron cristatum*

*^1^Sampling sites away from lakeshore. ^2^Soil electrical conductivity; data shown are mean and standard deviation values; different letters represent a significant difference among sites at 0.05 level.*

### Phytolith Extraction

Plant samples were sub-divided into aerial parts and roots, which were then washed with deionized water and ultrasonicated to remove impurities. The clean plant tissues were oven-dried at 70°C to a constant weight. A portion of the dried above-ground samples was cut into 2-mm pieces for phytolith extraction using a wet oxidation method modified from Parr and Sullivan (2014). Briefly, 750 mg of dried plant samples were digested in a Teflon digestion tube with 15 mL of concentrated nitric acid for 2 h at 150°C using a temperature-controlled graphite digestion apparatus (DigiBlock ED54, LabTech, China). Then, 5 mL concentrated nitric acid and 3 mL 30% hydrogen peroxide (H_2_O_2_) were added into the tube to oxidize the residual organic matter until only silica remained. The silica samples were then aggressively oxidized in boiling 30% H_2_O_2_ to ensure complete oxidation of the organic matter outside the phytoliths, and the samples were then rinsed to remove residual H_2_O_2_. The pure phytoliths were oven-dried at 75°C to a constant weight and stored for elemental analysis.

### Geochemical Analysis

Separate aliquots of the above-ground samples were ground using a micro grinding machine, and the dried phytolith samples were gently powdered in an agate mortar. Total Si and P contents were determined using molybdenum blue colorimetry with a UV spectrophotometer (UV-1800, Shimadzu, Japan). Sodium and potassium (K) contents in the plant samples were determined using inductively coupled plasma–optical emission spectrometry (ICP–OES 5110, Agilent, United States). Total contents of C and nitrogen (N) in plants and phytoliths were measured using an Elementar Vario EL III elemental analyzer (Elementar Analysensysteme, GmnH, Germany). Additionally, a field emission scanning electron microscope (FSEM) coupled with an energy dispersive X-ray spectrometer (EDS) (FESEM Sigma 500, ZEISS, Germany) was used to assess the morphological structure of phytoliths and the Si, C, and oxygen (O) composition of the surface of the phytoliths.

Air-dried soil samples were crushed to <2 mm. Soil pH and electrical conductivity (EC) were measured in a 1: 5 suspension of 5 g soil sample and 25 g deionized water using a pH meter and an EC meter, respectively. Soil bulk density was measured using a cutting ring method based on the weight of air-dried soil per volume sample. Soil moisture content was measured using a gravimetric method by comparative weighting of the fresh and oven-dried soil samples. Aliquots of the soil samples were ground to <0.15 mm in an agate mortar and bioavailable Si was extracted using 0.01 mol L^–1^ calcium chloride (CaCl_2_) solution according to a method modified from Hogan et al. (2018). Briefly, 1 g of dried soil sample was mixed with 30 mL CaCl_2_ solutions in 50 mL plastic centrifuge tubes. After being shaken for 16 h, the suspension was centrifuged, and the supernatant was separated to measure Si concentration using the colorimetric method described above.

### Statistical Analysis

All data were assessed using a one-way analysis of variance (ANOVA) followed by a Duncan’s test using SPSS version 17.0, where conditions of normality and homogeneity of variance were met. Linear regression analysis was conducted to determine the relationships between (a) plant Si and C content or accumulation; (b) plant salinity or Si contents and phytolith content, C in phytoliths, and PhytOC; and (c) soil bioavailable Si and plant Si storage. The correlations were determined using Pearson’s bivariate correlation analysis and a two-tailed *t*-test. All graphs were drawn using Origin version 8.0.

## Results

### Variations of Soil Parameters

Across the study area, soil pH differed significantly (*p* < 0.05) between different distances away from the lakeshores, with values of 10.1 ± 0.28 at the proximal sites (0–100 m) and 9.52 ± 0.40 at the distal sites (100–500 m). Soil EC value showed a similar decreasing trend as pH across the transects, with values of 2.66 ± 1.65 mS cm^–1^ at the proximal sites, and 0.90 ± 0.56 mS cm^–1^ at the distal sites. Similarly, soil moisture content significantly (*p* < 0.05) decreased from the proximal sites (12.8 ± 4.77%) to the distal sites (7.41 ± 3.44%). Bioavailable Si in soils, by contrast, increased from 128 ± 19.3 mg kg^–1^ at the proximal sites to 147 ± 17.8 mg kg^–1^ at the distal sites (Table 1). However, soil bulk density had no significant (*p* > 0.05) difference between proximal sites and distal sites. The different soil properties and habitats result in notably different plant communities (Table 1). The halophyte *Kalidium foliatum* grew near the lakeshores under a high salinity condition, with high soil pH and EC values but low bioavailable Si. The sites that are farther from the lakeshore are characterized by moderately salt-tolerant species, including *Leymus chinensis*, *Agropyron cristatum*, and *Arenariae radix* under conditions of lower salinity and higher soil bioavailable Si compared to the proximal sites. *Achnatherum splendens*, as a widely drought/salt-tolerant species, can survive in a wide range of habitats from low to high salinity levels and may be ubiquitous at all sites ranging from the proximal sites to the distal sites away from the lakeshores.

### Variations of Plant Elemental Compositions

The halophytes had considerably higher salinity (average Na^+^/K^+^ of 6.72 ± 3.00) and lower Si content (0.46 ± 0.26%) in their aerial parts compared to the moderately salt-tolerant grasses (Na^+^/K^+^ = 0.10 ± 0.08 and Si = 1.47 ± 0.63%) (Table 2). There was a significantly positive (*p* < 0.01) correlation between salinity and Si for moderately salt-tolerant grasses but a negative (*p* < 0.05) correlation for the halophytes (Table 3). Silicon content varied widely among moderately salt-tolerant grasses from 0.47% to 3.13% (dw). The Poaceae (i.e., *L. chinensis*, *A. splendens*, and *A. cristatum*) had higher Si content (average 1.52%) than that of Cyperaceae (i.e., *A. radix*) (average 1.10%). Plant C content ranged from 29.2% to 53.9%, with the lowest levels in *K. foliatum* (average 38%) and the highest in *A. splendens* (average 45%) (Table 2). Plant C content was significantly (*p* < 0.01) decreased by increased salinity in moderately salt-tolerant grasses, while salinity did not significantly (*p* > 0.05) affect C content in halophytes. A significant negative relationship between Si and C contents was observed for moderately salt-tolerant grasses (*p* < 0.05) but not for the halophytes (*p* > 0.05) (Table 3).

**TABLE 2:** Major elements and salinity level (Na^+^/K^+^ ratio) in plant samples and the mass ratios of elements between each other.

Species	Si%	C%	N%	P%	C/N	Si/C	Si/P	N/P	Na/K
*Kalidium foliatum* (*n* = 11)	0.46^a^ (0.26)	38.0^a^ (4.11)	2.08^a^ (0.30)	0.13^ab^ (0.02)	18.8^a^ (4.72)	0.01^a^ (0.01)	3.91^ab^ (2.42)	16.9^a^ (2.83)	6.72^a^ (3.00)
*Agropyron cristatum* (*n* = 8)	2.02^b^ (0.65)	42.9^b^ (0.48)	1.99^ab^ (0.41)	0.24^c^ (0.06)	22.4^a^ (4.84)	0.05^b^ (0.02)	8.73^a^ (3.10)	8.67^b^ (2.36)	0.17^b^ (0.09)
*Leymus chinensis* (*n* = 13)	1.72^b^ (0.64)	43.7^bc^ (0.60)	1.81^ab^ (0.30)	0.21^c^ (0.07)	24.7^ab^ (4.07)	0.04^b^ (0.01)	8.77^a^ (3.75)	9.23^b^ (2.61)	0.13^b^ (0.10)
*Arenariae radix* (*n* = 6)	1.10^c^ (0.24)	45.8^bc^ (4.28)	1.51^bc^ (0.37)	0.20^bc^ (0.01)	31.9^bc^ (7.82)	0.02^c^ (0.01)	5.39^ab^ (1.08)	7.41^b^ (1.86)	0.08^b^ (0.07)
*Achnatherum splendens* (*n* = 20)	1.19^c^ (0.49)	45.5^cd^ (1.60)	1.42^c^ (0.29)	0.09^a^ (0.04)	33.3^c^ (7.74)	0.03^c^ (0.01)	16.8^c^ (11.02)	18.8^a^ (8.17)	0.05^b^ (0.02)

*Result is expressed by average values of the same species at corresponding sampling site. The data in brackets is standard deviation. n is the number of plant samples. Different letters in superscript indicate a significant difference among plant species at 0.05 level.*

**TABLE 3:** Relationships between salinity (Na^+^/K^+^) and contents of Si, C, N and P for salt-tolerant grasses and the halophytic *K. foliatum.*

Samples	Elements	Na^+^/K^+^	Si	C	N	P
Salt-tolerant grasses (*n* = 47)	Na^+^/K^+^	1				
	Si	0.43**	1			
	C	−0.40**	−0.39**	1		
	N	0.15	0.26	–0.25	1	
	P	0.43**	0.36*	−0.37*	0.56**	1
Halophyte (*n* = 11)	Na^+^/K^+^	1				
	Si	−0.65*	1			
	C	–0.45	0.02	1		
	N	0.57	–0.31	–0.48	1	
	P	0.69*	–0.22	–0.54	0.61*	1

**Correlation at p < 0.01 and *Correlation at p < 0.05.

Although N content in both hylophytes and salt-tolerant grasses showed an increasing trend with salinity and was the highest in halophytes, there were no significant (*p* > 0.05) correlations between either Si or salinity and N content in grasses and halophytes. However, there was a significant positive correlation between Si and phosphorus (P) content for moderately salt-tolerant grasses, and a positive correlation of salinity and P content among the whole population of species (*p* < 0.05). Additionally, for all species, there was also a significant positive correlation between N and P content (*p* < 0.05) (Table 3).

### Silicon Uptake and Biomass Carbon Accumulation in Plants

**FIGURE 2:**
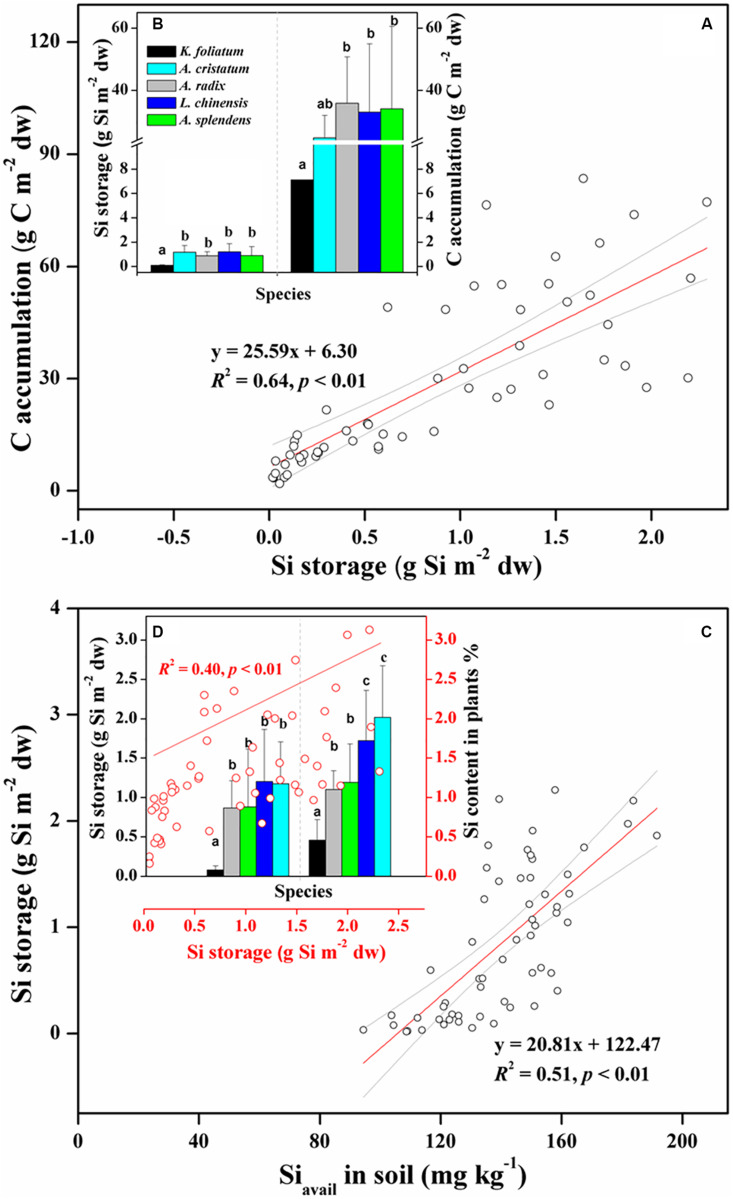
Positive relationship between plant Si storage and biomass C accumulation **(A,B)**; and positive effects of soil bioavailable Si on plant Si storage, and the further effects of Si storage on Si content in plants **(C,D)** among whole species including halophytes (*K. foliatum*) and moderately salt-tolerant grasses (*A. cristatum*, *A. radix*, *L. chinensis*, and *A. splendens*). Different letters represent a significant difference among plant species at 0.05 level, and the error bars represent the standard deviation of each species. Red lines represent significant correlations, and light gray line regions represent 95% confidence intervals.

To explore the relationship between Si storage and biomass C accumulation in plants under variable salinity conditions, we compared the capacity of Si storage and amount of biomass C between the halophytes and the salt-tolerant grasses. The total amounts of Si storage in aboveground portions of each species in one quadrat (1 m × 1 m) showed a significant (*p* < 0.01) correlation with those of C (Figure 2A). As shown in Figure 2B, the halophytes stored the least Si and C, with average values of 0.08 ± 0.05 g Si m^–2^ dw and 7.10 ± 4.01 g C m^–2^ dw, respectively. In the salt-tolerant grasses, the Poaceae including *L. chinensis*, *A. splendens*, and *A. cristatum* stored more Si (1.04 ± 0.68 g Si m^–2^ dw) than the Cyperaceae *A. radix* (0.87 ± 0.34 g Si m^–2^ dw), which showed the same trend with plant Si content (Figure 2D). There was also a significant (*p* < 0.01) positive correlation between soil bioavailable Si and plant Si storage for all of plants from different habitats with variable soil pH and EC conditions (Figure 2C and Table 1). In contrast, *A. radix* supports higher biomass C accumulation (35.9 ± 14.9 g C m^–2^ dw) than *L. chinensis*, *A. splendens*, and *A. cristatum*, which was mainly due to high C content holding in *A. radix* (Figure 2B and Table 2). Furthermore, due to its variably salt-tolerant adaptation, *A. splendens* exhibited a large variation in biomass C accumulation from 3.60 g C m^–2^ dw to 83.5 g C m^–2^ dw with the mean value of 34.1 ± 26.5 g C m^–2^ dw among different quadrats.

### Phytolith C Sequestration in Plants

**FIGURE 3:**
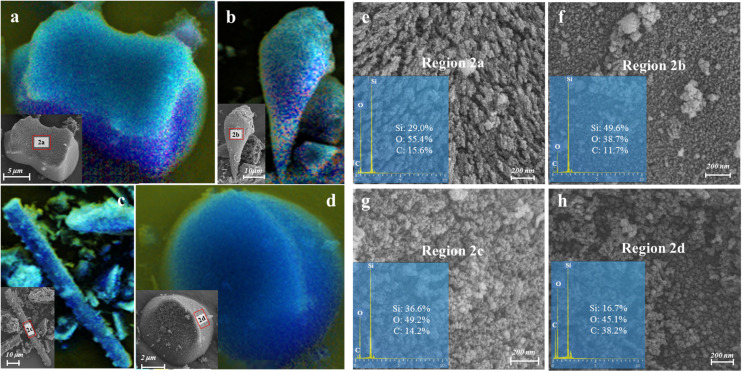
Images of various morphologies and the mapping of major element distribution (i.e., blue shading for Si, green shading for O and yellow shading for C) on the surface of the phytoliths extracted from *A. splendens* **(a,b)** and *A. cristatum* **(c,d)** characterized by field-emission scanning electron microscope combined with Energy-dispersive X-ray spectroscopy (FSEM-EDS). **(e–h)** were the nano-scale (200 *nm*) structure magnified by SEM and the mass percent of Si, O, and C assessed by EDS spectra on the phytolith surface obtained at regions 2a, 2b, 2c, and 2d, respectively.

**FIGURE 4:**
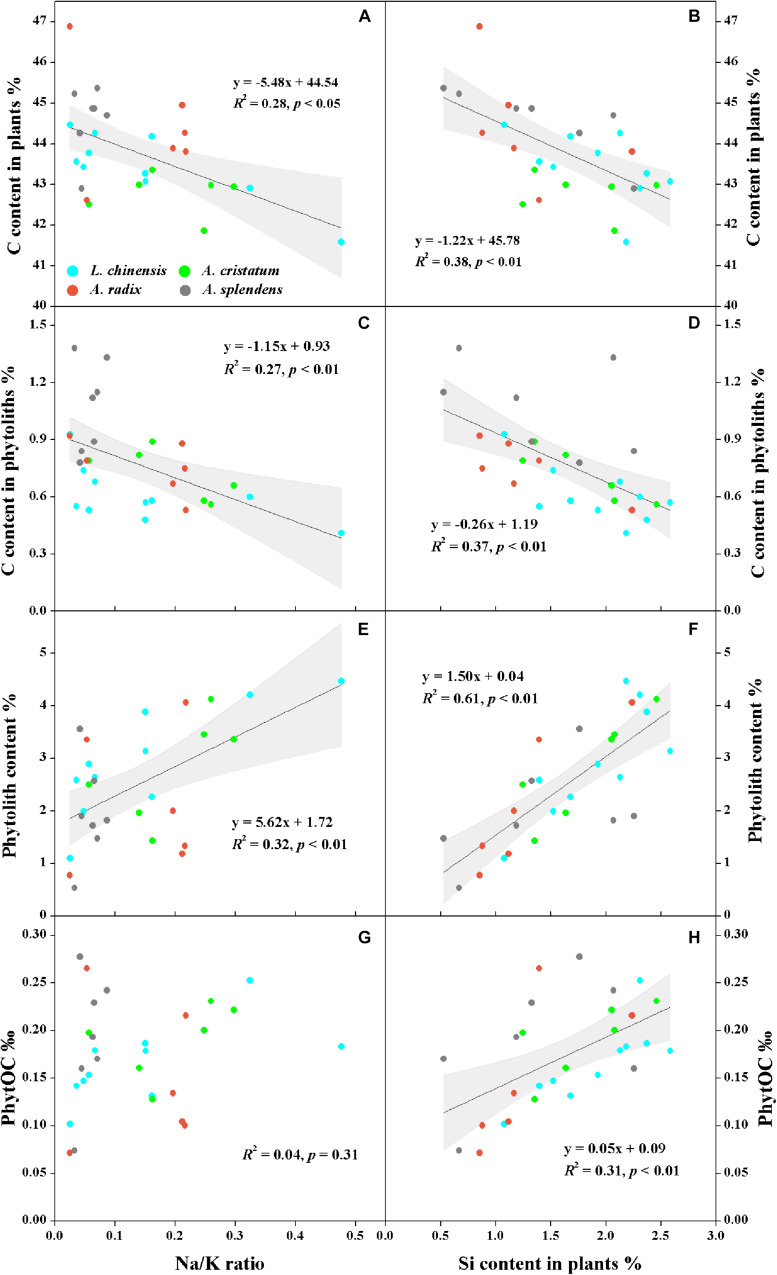
Relationships between plant salinity level (Na/K ratio) or silicon content and carbon in plants **(A,B)** and phytoliths **(C,D)**, phytolith content **(E,F)**, and PhytOC **(G,H)** of moderately salt-tolerant grasses. Dark gray lines represent significant correlations, and light gray regions represent 95% confidence intervals.

The qualitative and semi-quantitative analysis of phytoliths by SEM/EDS showed that the major elements in phytoliths were Si and O, which accounted for more than 80% of the elements distributed on the surfaces of most phytoliths (Figures 3a–d). There was a small amount of C (10% ∼ 20%) inserted into the silica structure (Figures 3e–g), and the observation of relatively high C distribution (Figure 3h) may result from the loosely arranged amorphous silica nano-particles that adsorb more C compounds than the compacted silica structures on phytolith surfaces. Local magnification of each phytolith showed that different phytolith morphotypes were formed by different sizes of silica nano-particles, resulting in different compactness of the phytolith and different amounts of occluded-C in the phytoliths (Figures 3e–h). In addition, we had aimed to investigate the effects of plant salinity and Si on the phytoliths and PhytOC, but there was no phytolith extracted from halophytes because of their low Si uptake and phytolith formation. In salt-tolerant grasses, C contents of both plants and phytoliths were significantly negatively correlated with plant Si content and salinity levels (Na/K ratio); while phytolith contents were positively correlated with plant Si content and salinity level (*p* < 0.01) (Figure 4). The carbon content (average value of 0.77%) of dry phytoliths varied greatly among the grasses, with the highest values in *A. splendens* (1.07%) and the lowest in *L. chinensis* (0.61%). PhytOC content also varied among the grasses (ranging from 0.07 to 0.28‰ of plant dry weight) while showing a significant positive correlation with Si content (*p* < 0.01) (Figure 4H); however, there was no correlation with salinity levels (Figure 4G).

## Discussion

### Response of Plant Silicon and Carbon to Salinity

Since plant Si uptake is driven by osmotic and hydraulic processes, low soil water availability and high salt ion content can decrease Si uptake. However, the Si-accumulators can maintain high Si uptake, given sufficient Si supply, even under adverse climatic conditions (Issaharou-Matchi et al., 2016). In the current study, soil bioavailable Si (Si_*avail*_) decreased with increasing soil pH (from 9.52 to 10.1), which is consistent with the previous report by Berthelsen et al. (2003) that an increase in soil pH decreased CaCl_2_ extractable soil Si (Table 1). Haynes (2019) reported that high soil pH increased adsorption of H_4_SiO_4_ onto metal-oxide surfaces and the maximum adsorption was at pH 9 to 10, thus resulting in the decrease of Si mobility and availability in soils. That report also observed that increased soil pH causes enhancement of phytolith solubility and therefore leads to a short-term increase of bioavailable Si releasing into soil solutions (Klotzbücher et al., 2018a). However, there was a reduction in the biogenic Si pool in topsoil over the longer term due to the strong leaching ability of a sandy soil in arid and semi-arid grasslands.

Plant Si content was positively correlated with salinity level in salt-tolerant grasses and negatively correlated with salinity level in halophytes (Table 3), indicating that the relatively high temperature caused high transpiration, facilitating ion transport and active Si uptake for the Si-accumulators (Coskun et al., 2016). Furthermore, the positive relationships between soil bioavailable Si, plant Si storage and Si content resulted in a higher Si content in moderately salt-tolerant grasses than that in halophytes (Figures 2C,D). In addition, low Si accumulation, and the negative correlation between plant Si and salinity in halophytes (Table 3) indicated a non-Si-dependent salt-tolerance mechanism. Halophytes usually maintain high osmotic potential in order to take up water, by which they accumulate sufficient inorganic ions (e.g., Na^+^ and Ca^2+^) and decrease the uptake or acropetal translocation of Si or other elements via salt ion competition (Moghaieb et al., 2004; Hu and Schmidhalter, 2005).

**FIGURE 5:**
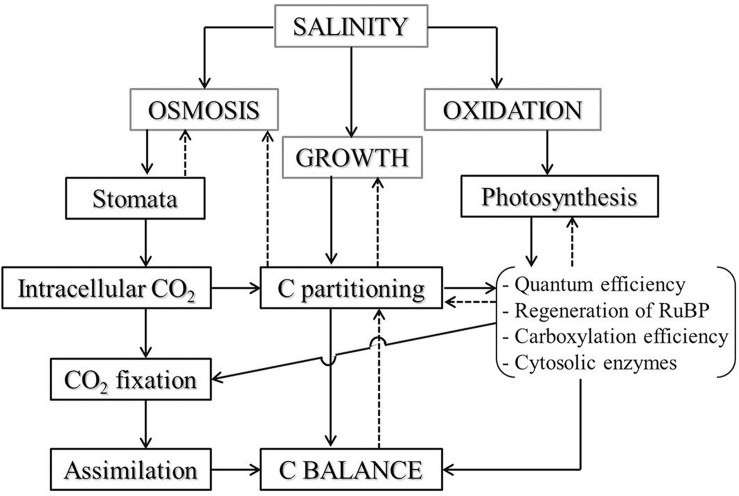
Salinity effect on CO_2_ assimilation and plant C balance via limitation of stomata and photochemical metabolism in plant leaves. When plants suffering from salinity stress, osmotic and oxidative stress would be induced and may lead to stomatal alteration and photosynthetic inhibition, restricting CO_2_ flux into plants and damaging photosynthetic apparatus, and thereafter affecting C partitioning in plant tissues and disturbing assimilation process. The altered C balance, in turn, affects plant growth through disturbing C partitioning and photosynthesis. The solid arrows represent the chain reactions during the process of salinity influence on plant C balance and the dashed lines with arrows represent the feedback relations, but these direct reactions and the feedbacks always occurred simultaneously. The sketch was modified from Chaves (1991).

Although environmental conditions such as temperature and moisture affect terrestrial biogeochemical cycles (Shaw et al., 2002; Song et al., 2012b; Zhang et al., 2012), the role of salinity on coupled biogeochemical cycles of Si and C in grasslands *in situ* is still not well understood (Rios et al., 2017). In this study, reduction of plant C content, along with salinity increase in the salt-tolerant grasses growing in the natural field, might result from the nutrient imbalance and growth inhibition associated with excessive Na^+^ accumulation in plants under salinity stress. Chaves et al. (2009) reported that salinity caused osmotic stress and water depletion in plants, and therefore altered C fixation via the closure of stomata in leaves. Furthermore, salt ion accumulation in plants affects photosynthetic CO_2_ assimilation (Meloni et al., 2003) by restricting CO_2_ diffusion toward the chloroplast and C metabolism in photosynthesis (Chaves, 1991; Figure 5). Moreover, the increase of N contents in response to salinity caused a reduction of C/N ratio, which partly indicates the inhibition of photosynthesis and plant growth that resulted from salt ion accumulation in the grasses (Johnson and Hartley, 2018). There is, however, an important role of leaf N content about photosynthetic C fixation and plant biomass accumulation, during which environmental change and species difference are responsible for influencing the stoichiometry of C and N in plants (Sardans et al., 2008).

### Silicon Regulation of Phytolith/PhytOC Production

Previous researchers have reported that drought and high evapotranspiration rates would increase Si accumulation and cell silicification in plant leaves (Issaharou-Matchi et al., 2016), but few studies have investigated salinity effects on phytolith formation in Si-accumulators. Moreover, a recent study by Johnson et al. (2019) found that warming combined with Si supplementation increased Si accumulation in the leaves of *Phalaris aquatica* by 24.0%, relative to ambient temperature. A study by Meunier et al. (2017) suggested that plants might accumulate phytolith over veins to strengthen leaf structure and enhance light interception and photosynthesis under drought stress. These reports are supported by our observations because plants suffer similar physiological water deficit under salinity stress as they do in drought conditions (Chaves et al., 2009). However, further research on the assessment of species-specific mechanisms of Si or phytolith in response to salinity is necessary due to the specific ionic toxicity and osmotic stress induced by salinity.

When plants take up H_4_SiO_4_ from soil solutions, silica can be transported along the transpiration stream and deposited in plant tissues forming silica gel nano-particles, and developed eventually into various types of phytoliths (Li et al., 2017; Figure 3). Phytolith microstructure and surface element composition characterized by SEM/EDS indicated that differently sized nano-particle silica should control various phytolith shaping and PhytOC content. We hypothesize that compactness and specific surface area of phytoliths may be closely related to plant Si content and transpiration rate, and these characteristics of phytoliths can affect the capacity for C occlusion within the phytoliths (Li et al., 2013). Besides, it has also been reported that phytolith development in the leaves of Si-accumulating plants increased with Si uptake as well as water evaporation (Li et al., 2017). In the present study, the grasses are considered to have high phytolith contents in the late stage of growing season with relatively high evaporation (Klančnik et al., 2014). Therefore, we suggest that this is one of the critical stages to investigate long-term C cycles because of the most amount of PhytOC being sequestrated in grass leaves at the mature stage of plant growth. However, knowledge gaps remain and require further research on the differences of PhytOC production in plants between different growth stages. In addition, the positive correlation of PhytOC and Si content demonstrates that Si-promoted phytolith production generally enhances C encapsulation despite the decreased C and increased Si content in phytoliths. However, there was no significant correlation between salinity and PhytOC, regardless of the positive correlation between salinity and phytolith content in salt-tolerant grasses (Figure 4). This may result from the inhibition of CO_2_ assimilation and C fixation by salinity stress, leading to a reduction of C accumulation in plants as well as in phytoliths (Zuo et al., 2017). Therefore, our results indicate that PhytOC content in plants mainly depends on Si content but is also impacted by environmental factors. Nonetheless, to identify the effects of Si on long-term C cycles, further studies such as investigating the stable isotopes of C and Si in plants as well as in phytoliths, and more specific investigation of the morphology of phytoliths are required to improve the understanding of the relationship between environmental changes and phytolith production dynamics (Geilert et al., 2014).

### Silicon Effect on Plant Carbon Under High Salinity Conditions

**FIGURE 6:**
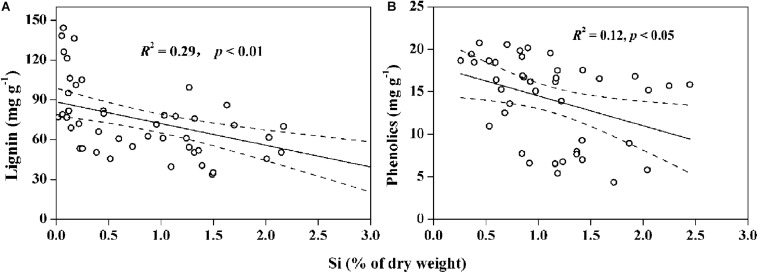
Negative correlations between **(A)** content of silicon (Si) and lignin in wetland plants (data collected from Schoelynck et al., 2010; Schaller et al., 2012; Suzuki et al., 2012), and **(B)** content of Si and phenolics in dryland plants (data collected from Frew et al., 2016; Johnson and Hartley, 2018). Solid lines represent the significant correlations, and the dashed line regions represent 95% confidence intervals.

Increasing soil bioavailable Si can enhance the competitiveness of Si-accumulating grasses while maintaining or improving productivity of the grasslands in arid and semi-arid regions (Etesami and Jeong, 2018). Our results show significantly (*p* < 0.01) increased Si uptake in plants with the increase of bioavailable Si in soils (Figure 2). Consequently, plant C accumulation increased with Si uptake in salt-tolerant grasses and halophytes, suggesting an improvement of plant photosynthesis and biomass C fixation by Si storage. For halophytic species, Si-activated H-ATPase in the root plasma membrane can decrease salt stress by enhancing K^+^ transport relative to Na^+^ from plant roots to aerial parts through the apoplastic pathway (Gong et al., 2006; Mateos-Naranjo et al., 2013). For Si-accumulators, silica deposition in plant tissues substitutes for lignin components to strengthen cell walls, improving leaf erectness or light capture and enhancing plant growth under stressed conditions (Epstein, 1994; Detmann et al., 2012). Recently, some researchers have pointed out a defense trade-off strategy between Si and C-based structural components (i.e., phenolic, lignin, or cellulose) in some Si-accumulators, such as *Oryza sativa*, pasture and wetland grasses (Schoelynck et al., 2010; Johnson and Hartley, 2018; Klotzbücher et al., 2018b; Figure 6). These studies reported that Si substitution of C for biosynthesizing structural components expends lower energy than the energy-costly lignin metabolism, and the energy saved may help to maintain plant biomass in adverse environments. The significantly negative relationship between Si and C contents in moderately salt-tolerant grasses, shown in Figure 4B, is consistent with previous studies of Poaceae grasses or cereals (Schaller et al., 2012; Neu et al., 2017), thus providing additional evidence for the defense trade-off strategy as well as Si regulation of plant growth and biomass C accumulation under environmental stresses.

**FIGURE 7:**
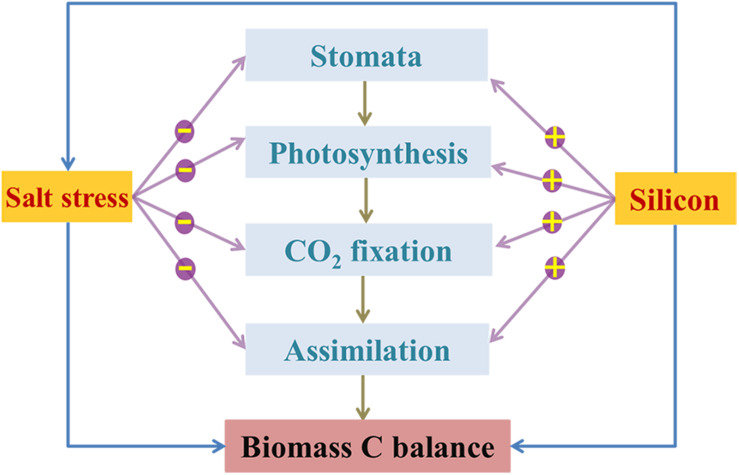
Schematic sketch for the limitation (negative) of salinity stress on plant C balance via inhibiting photosynthetic C metabolism, and the alleviation (positive) of Si on salt toxicity to plant by indirectly improving photosynthetic CO_2_ assimilation and thus enhancing plant biomass C.

Since different strategies for coping with environmental stresses among various species result in different trade-offs between Si and C-based structural components (Schoelynck et al., 2010), understanding relationships between Si, salinity, and C in plants is essential for revealing the mechanisms by which Si enhances plant biomass under salinity stressed conditions (Zuo et al., 2014). In this work, there was low Si content in halophytes and no correlation between the contents of Si and C (Table 3), mainly due to the passive Si uptake and lower Si demand of these plants. Based on the lower Si content in *K. foliatum* than other species that measured in our study and also a report by Liang et al. (2006), *K. foliatum* takes up Si passively or excludes Si from its tissues, resulting in low silica deposition and substitution for organic C during cell growth. In fact, as one kind of the chenopodiaceae halophytes, *K. foliatum* can maintain its osmotic balance through increasing accumulation of carbohydrate, especially free proline and soluble sugars. These outcomes indicate that the metabolic C flux in these species is mainly used to build the C-based skeleton in plant tissues under high salinity conditions (Rosa et al., 2009). However, Si can bind to cellulose molecules through hydrogen bonding in cell walls of Si-accumulating species, resulting in an inverse correlation between the contents of phenolic compounds and Si in plant leaves (Klančnik et al., 2014). Schoelynck et al. (2010) reported that plant species containing high Si more readily incorporate abundant Si as structural components at a lower energetic cost than Si-excluded species, which may partly explain the higher biomass C accumulation of grasses than halophytes in this field investigation. Moreover, the lower energy-cost of defense acquisition by Si than that by C-based compounds during plant growth can cause interspecific differences in biomass C accumulation in plants under different conditions (Schoelynck et al., 2012). This likely explains why *A. splendens*, in our study sites, exhibited a considerable variation in biomass C accumulation among different quadrats. In general, the relationship between Si and C in plants is highly dependent on species as well as the growth surroundings, and both Si and salinity can act as a driver for ecological changes and phytogenesis (Liang et al., 2006; Bui, 2013). Thus, we propose a general schematic sketch for the limitation of salinity stress and the mediation of Si on plant biomass C balance (Figure 7). However, further investigation into the effect of environmental factors on soil bioavailable Si and the response of Si to toxic elements (i.e., Na, Cd, and As, etc.) in plants would facilitate the development of more effective strategies for improving Si availability and plant growth.

## Conclusion

Salinity stress as one of the major abiotic stresses can not only alter plant communities but also decrease productivity, affecting element biogeochemical cycles in arid and semi-arid grasslands. In this work, soil bioavailable Si showed great potential to enhance plant growth and C fixation through enhancing plant Si storage in the grassland ecosystems under high-salinity conditions. The significantly negative relationship between Si and C content for moderately salt-tolerant grasses compared to the absence of such a relationship in halophytes provides a novel case study of the trade-off strategy of Si- and C-based structural compounds in Si-accumulator dominant ecosystems under environmental stresses. These results also imply that Si played a more important role for salt-tolerant Si-accumulators in regulating biomass C accumulation as well as long-term C sequestration in the form of PhytOC than for halophytes. However, further experiments assessing the levels of Si in plant structural tissues among various species under different environmental conditions should be conducted to better understand the effects of Si on C cycling and CO_2_ assimilation in soil–plant systems.

## Data Availability Statement

All datasets generated for this study are included in the article/supplementary material.

## Author Contributions

LL and ZS conceived the ideas and designed the methodology. LL conducted the laboratory work and statistical analysis with directions from CY and BS. RE, GY, HL, and HW helped in the writing of the manuscript. All authors contributed critically to the drafts and gave final approval for publication.

## Conflict of Interest

The authors declare that the research was conducted in the absence of any commercial or financial relationships that could be construed as a potential conflict of interest.
